# Diagnostic outcomes of radial endobronchial ultrasound bronchoscopy guided by manual navigation in the evaluation of peripheral pulmonary lesions: An observational study

**DOI:** 10.1111/crj.13768

**Published:** 2024-04-29

**Authors:** Mingli Yuan, Yi Hu, Liangchao Wang, Wen Yin, Yang Xiao

**Affiliations:** ^1^ Department of Pulmonary and Critical Care Medicine, Central Hospital of Wuhan, Tongji Medical College Huazhong University of Science and Technology Wuhan China

**Keywords:** interventional pulmonology, manual navigation, navigational bronchoscopy, peripheral pulmonary lesion, radial endobronchial ultrasound

## Abstract

**Background and Aims:**

Manual navigation (MN), drawing a bronchoscopic road map simply by looking at the consecutive computed tomography (CT), is feasible and economical. However, scant data about the use of MN in radial endobronchial ultrasound (r‐EBUS) bronchoscopy have been documented till now. We aimed to evaluate the diagnostic performance of r‐EBUS bronchoscopy guided by MN for diagnosing peripheral pulmonary lesions (PPLs) and to determine clinical factors affecting the diagnostic yield.

**Methods:**

We performed a retrospective, cohort study of consecutive patients with PPLs who underwent r‐EBUS bronchoscopic biopsy via guidance of MN from May 2020 to June 2021 in our Respiratory Endoscopic Division. The overall diagnostic yield of MN‐guided r‐EBUS, the factors affecting the yield, and the diagnostic performance for malignancy were evaluated.

**Results:**

A total of 102 patients (103 lesions) were evaluated. The overall diagnostic yield of MN‐guided r‐EBUS was 82.0%, and it ranged from 79.6% to 82.5%, assuming the undermined cases were all positive cases (79.6%) or negatives (82.5%). The sensitivity of MN‐guided r‐EBUS for malignancy was 71.4%, ranging from 68.2% to 71.4%, the specificity was 100%, the positive predictive value was 100%, and the negative predictive value was 67.3%, ranging from 63.8% to 69.0%. The multivariate logistic regression showed that “bronchus sign on CT” was the only predictor of the overall diagnostic yield (odds ratio = 11.5, 95% confidence interval: 1.9–70.9, *P* = 0.009).

**Conclusions:**

MN‐guided r‐EBUS is feasible in diagnosing PPLs, especially for lesions with bronchus sign on CT.

## Graphical abstract

Manual navigation (MN), rolling and flipping the CT images to correlate with the orientation in bronchoscopic view and then drawing a bronchoscopic road map at every bronchial bifurcation at precisely the same angle with the flipped/rotated CT showed, is an efficient navigation method in radial endobronchial ultrasound (r‐EBUS) bronchoscopy for diagnosing PPLs.

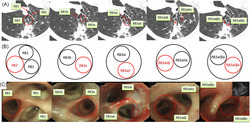

**Abbreviations**
CIs: confidence intervals
CT: computed tomography
ENB: electromagnetic navigation bronchoscopy
GS: guide sheath
MN: manual navigation
ORs: odds ratios
PPLs: peripheral pulmonary lesions
r‐EBUS: radial endobronchial ultrasound
ROSE: rapid on‐site evaluation
VBN: virtual bronchoscopic navigation

## 1 INTRODUCTION

Because of the results of the National Lung Screening Trial,^1^ low‐dose chest computed tomography (CT) screening for lung cancer has become a standard of care, and thus, increased peripheral pulmonary lesions (PPLs) that require histological confirmation have been detected. CT‐guided percutaneous transthoracic needle biopsy has been widely adopted for diagnosing PPLs. Despite a good sensitivity of 94%,^2^ the accompanied high complication rate, which was reported to be 18.8–25.3% risk of pneumothorax and 6.4–18% risk of pulmonary hemorrhage,^3^ restricted its use in sampling PPLs. Conventional bronchoscopy has a sensitivity for malignancy ranging from 34% to 63% in diagnosing PPLs.^4^ Though radial endobronchial ultrasound (r‐EBUS) has improved the diagnostic sensitivity to 72% according to a recent large meta‐analysis,^5^ it is still far from enough. One of the difficulties in sampling PPLs by r‐EBUS bronchoscopy is how to find the road to the lesion. Various bronchoscopic navigation devices have been developed to reach PPLs, such as virtual bronchoscopic navigation (VBN), electromagnetic navigational bronchoscopy (ENB), and even robotic‐assisted bronchoscopy.^6^ They indeed bring increased diagnostic yields, while they are expensive, time‐consuming, and impossible to be available in all bronchoscopy centers.

Manual navigation (MN), drawing a schematic representation of the bronchial branching to reach the PPLs simply by looking at the consecutive CT, requires only paper and pencil.^7^ This method is not only economical but also brings a high navigation success rate and an increase diagnostic yield in PPLs.^8^, ^9^, ^10^ Unfortunately, scant data about this method have been documented till now.

Therefore, we aimed to evaluate the diagnostic performance of r‐EBUS bronchoscopy guided by MN for diagnosing PPLs and malignancies and to determine clinical factors affecting the diagnostic yield.

## 2 MATERIAL AND METHODS

### 2.1 Study design and participants

This single‐center, retrospective, cohort study consecutively enrolled patients with PPLs who underwent r‐EBUS bronchoscopic biopsy from May 2020 to June 2021 in our Respiratory Endoscopic Division. PPLs were defined as the lesions invisible during the routine bronchoscopy. Inclusion criteria are as follows: (1) patients >18 years old with PPLs who underwent r‐EBUS bronchoscopic biopsy; (2) signed the consent form of bronchoscopic biopsy; (3) patients had no bronchoscopy contraindications; and (4) procedures guided by the MN. Exclusion criteria are as follows: (1) patients who underwent rebiopsy for molecular and/or immune testing after chemotherapy and/or immunotherapy; (2) r‐EBUS bronchoscopic biopsy for diagnosis of diffuse interstitial lung disease; (3) procedures involved in the use of other navigation method, such as VBN or ENB; (4) abnormal lumen indicated by routine bronchoscopy; and (5) undetermined cases without 6 months follow‐up.

### 2.2 Manual navigation

All patients underwent thin slice (<1 mm) chest CT for MN. Y. X. and M. L. Y. draw the sketches independently, and agreement was reached after discussion. The steps were as follows.

First, identify the target bronchus. Carefully read the CT images, axial views were mainly referred to, and coronal and sagittal views were as supplementary. Rolled the CT images slowly from the segmental bronchus to find the leading bronchus to the lesion; if the peripheral bronchus was too small to be identified, the accompanying vessels might be guided. The furthest bronchus or vessels that could be recognized were the target.

Second, determine the orientation in regular bronchoscopic view. Because of the reversal and rotation of the bronchoscope, the orientation for different lung lobes in bronchoscopic view was different. It was important to know exactly the relative spatial position (cranial–caudal, ventral–dorsal, and mediastinal–lateral) in the regular bronchoscopic view. Then, rolled the CT images to correlate with the orientation in bronchoscopic view. The methods for flipping and rotating CT images were listed below.

**FIGURE 1:**
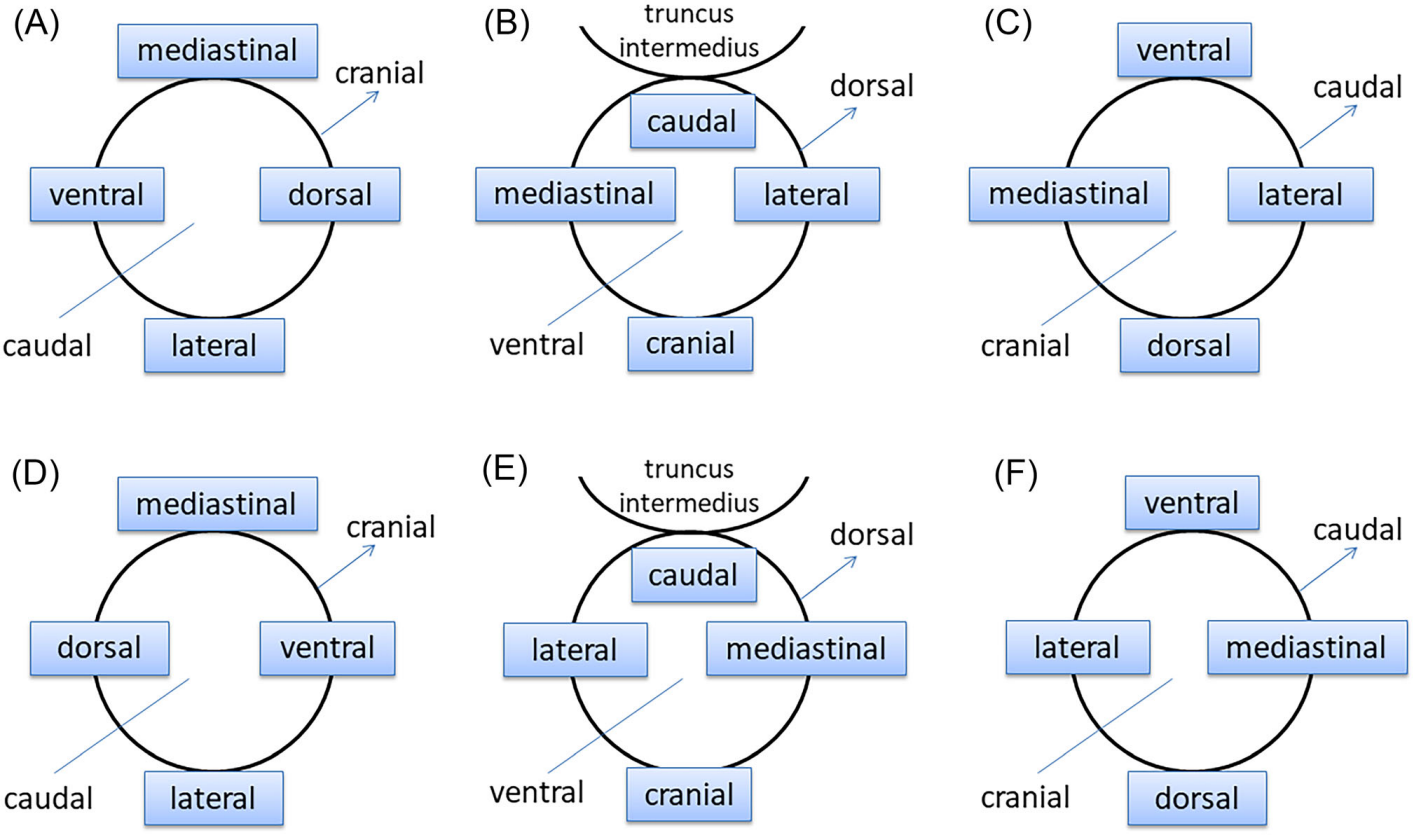
The orientation in regular bronchoscopic view. (A) For right upper lobe, the top of the bronchoscopic view is mediastinal, the bottom is lateral, the left is ventral, the right is dorsal, the proximal is caudal, and the distal is cranial. The orientations in bronchoscopic view for right dorsal segment, right middle lobe and lower basal segment, left superior segment, left dorsal segment, and left lingular segment and lower basal segment, respectively (B–F).

*The right upper lobe*: The orientation in regular bronchoscopic view was shown in Figure 1A; thus, the axial CT images would be rotated counterclockwise 90°.
*The right dorsal segment*: The bronchus moves in the caudal direction, and then, RB6a turns to the cranial direction. If the bronchoscope looked down from the truncus intermedius, the orientation in bronchoscopic view was shown in Figure 1B. Thus, the axial CT images would be reversed left to right, and the cranial–caudal direction should be drawn upside‐down in the sketches.
*The right middle lobe and lower basal segment*: The orientation in regular bronchoscopic view was shown in Figure 1C; thus, the axial CT images would be reversed left to right.
*The left superior segment*: The orientation in regular bronchoscopic view was shown in Figure 1D; thus, the axial CT images would be rotated clockwise 90°.
*The left dorsal segment*: If the bronchoscope looked down from the truncus intermedius, the orientation in bronchoscopic view was shown in Figure 1E. Thus, the axial CT images would be reversed right to left, and the cranial–caudal direction should be drawn upside‐down in the sketches.
*The left lingular segment and lower basal segment*: The orientation in regular bronchoscopic view was shown in Figure 1F; thus, the axial CT images would be reversed right to left.

Third, draw the sketches at every bronchial bifurcation at precisely the same angle with the flipped/rotated CT showed. It was important to keep the sketches at hand and rotate it with the angle of bronchoscope rotated during the operational process.

**FIGURE 2:**
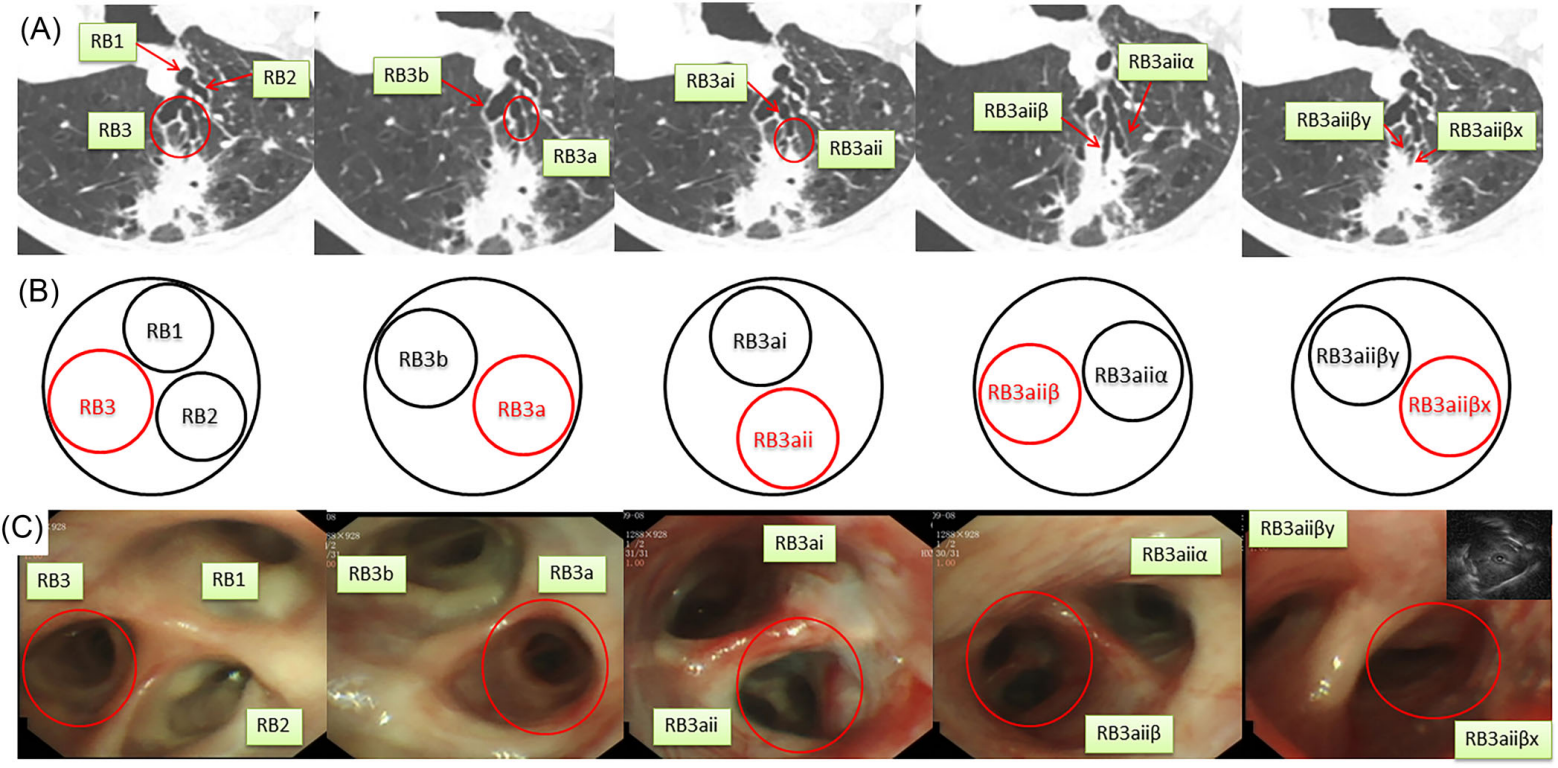
A representative case of right upper lobe lesion. (A) Rolled the computed tomography images counterclockwise 90° to correlate with the orientation in bronchoscopic view. (B) Drew the sketches at every bronchial bifurcation at precisely the same angle with the rotated computed tomography showed. (C) The sketches were exactly the same as the actual bronchoscopic views, and within radial endobronchial ultrasound image was identified at target RB3aiiβx. This patient was diagnosed as tuberculosis by radial endobronchial ultrasound bronchoscopy.

A representative case was shown in Figure 2.

### 2.3 Procedures

Three experienced bronchoscopists (Y. X., L. C. W., and Y. H.) completed the procedures under moderate sedation with the combination of midazolam and sufentanil or general anesthesia with a laryngeal mask airway according to the bronchoscopists. A thin scope (P260F or P290; Olympus) (4.0 or 4.2 mm outer diameter with a 2.0 mm working channel) or a thick scope (1TQ290; Olympus) (5.9 mm outer diameter with 3.0 mm working channel) was first used to examine the airway, and if there were no visible abnormalities, the bronchoscope was advanced into the most distal part of the target bronchus according to the simulated map. After that, the r‐EBUS probe (UM‐S20‐17S or UM‐S20‐20R; Olympus) with a guide sheath (GS) (K‐201 or K‐203, Olympus) was inserted to detect the lesion and then kept the GS in place and removed the probe. r‐EBUS findings were categorized as within, adjacent to, or invisible according to the positional relationship between the probe and target PPLs.

For transbronchial forcep, general anesthesia with endotracheal intubation was adopted, and 1.9 or 1.5 mm forceps were introduced into the GS to biopsy. For transbronchial cryobiopsy, a block balloon was placed at the ostium of target segmental bronchi in advance, and the 1.1 mm outer diameter cryoprobe (ERBECRYO2, ERBE) was inserted into the GS, activated for 5–7 s, and removed with the GS and bronchoscope together from the airway. Termination of operation was determined by referring to the result of rapid on‐site evaluation (ROSE). The biopsied specimen was fixed in formalin for pathological diagnosis, and the GS flushing fluid was sent to cytological and/or microbiological examination.

Post procedure, a chest radiograph was performed routinely to check for pneumothorax.

The biopsy strategy was used at the operator's discretion and proficiency to the technology. In general, cryobiopsy was less‐frequently used because of the complexity and high risk of hemorrhage of this procedure, but for lesions located in the distal bronchus and with “adjacent” features in r‐EBUS findings, if the operator was quite skilled, cryobiopsy was preferred.

### 2.4 Outcome measures

Demographic data of patients, imaging characteristics of the lesions, process of the bronchoscopy, complications of procedure, diagnosis of r‐EBUS biopsy, and the final diagnosis were recorded. The airway generation in this study was defined according to the Japanese classification.^7^ The main bronchi were designated as Generation 0, the lobar bronchi as Generation 1, segmental bronchi as Generation 2, and so forth. Complications associated with bronchoscopy were recorded. According to standardized definitions of bleeding,^11^ hemorrhage was graded as follows: (1) mild: blood suctioning required for <1 min; (2) moderate: suctioning required for >1 min, repeat wedging of the bronchoscope for persistent bleeding needed, or the application of cold saline, diluted vasoactive substances, or thrombin; (3) severe: selective intubation needed for <20 min; and (4) life‐threatening: persistent selective intubation or emergency care required.

If the diagnosis made by the pathology and/or microbiology of specimens harvested by bronchoscopic biopsy accorded with the final diagnosis, they were defined as true positive cases (malignancy) or true negative cases (nonmalignancy). The final diagnosis was made by comprehensive analysis of clinical characteristics, bronchoscopy and/or surgery results, and reexaminations during follow‐up. Given the small amount of specimen obtained by bronchoscopy and the heterogeneity of cancer cells in the tissue, if bronchoscopy revealed “nonspecific inflammation,” a definite diagnosis then could not be made, and a final diagnosis of chronic inflammation would be made only when the PPLs reduced in size or disappeared after ≥6 months of follow‐up. Similar definition was seen in the previous study.^12^ For PPLs that had grown or remained unchanged, the diagnosis was unknown. Overall diagnostic yield was defined as true positive cases and true negative cases out of the number of PPLs underwent bronchoscopy and had definite diagnosis. Undiagnosed cases were included in a sensitivity analysis, assuming all as true negative or false negative cases. This provided low and high estimates of the diagnostic yield, sensitivity, specificity, positive predictive value, and negative predictive value of diagnosing malignancy.

### 2.5 Statistical analysis

Data analysis was performed with SPSS (version 22, Chicago, IL, USA). Continuous variables were presented as means and standard deviations, and categorical variables were presented as absolute numbers and percentages. Categorical variables were compared between the groups using the Fisher exact test. Univariate and multivariate analyses were performed using logistic regression models to determine the factors associated with the diagnostic yield. Baseline variables that were considered clinically relevant or that showed a univariate relationship with outcomes with *P*‐values less than 0.2 were entered into multivariate logistic regression model. No covariates included in the regression models had missing values, and no variables included in the multivariate regression model showed collinearity. The odds ratios (ORs) and 95% confidence intervals (CIs) were calculated. A value of *P* < 0.05 was considered statistically significant.

## 3 RESULTS

### 3.1 Baseline characteristics of included patients, target lesions, and procedures

**FIGURE 3:**
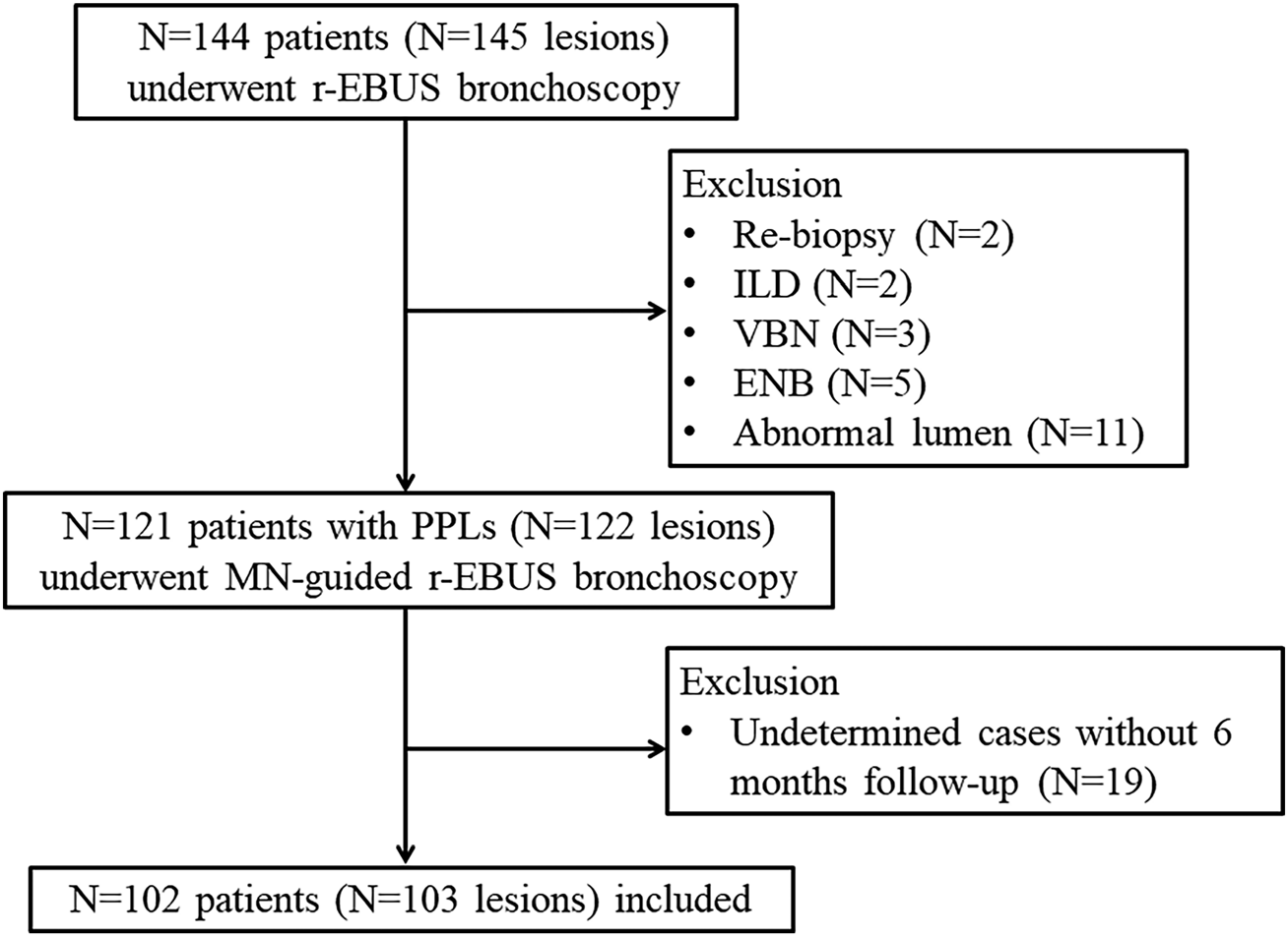
Flow diagram of the study population. ENB, electromagnetic navigation bronchoscopy; ILD, interstitial lung disease; MN, manual navigation; PPL, peripheral pulmonary lesion; r‐EBUS, radial endobronchial ultrasound; VBN, virtual bronchoscopic navigation.

As shown in Figure 3, a total of 145 r‐EBUS procedures (144 patients) were performed during the study period. Twenty‐three cases were excluded: Two cases were tumor patients who received rebiopsy, two cases were biopsied to confirm the classification of interstitial lung disease, three and five cases were guided by VBN and ENB, respectively, and 11 cases were found to have abnormal lumen during the bronchoscopy. For analyzing the final diagnostic yield, a total of 102 patients (103 lesions) were evaluated after excluding 19 undetermined cases without 6 months of imaging follow‐up.

Table 1 summarizes the baseline characteristics of included patients with PPLs. The mean size of the targeted lesions was 40.8 ± 21.7 mm, with lesions >20 mm accounting for 79.682.5% of the lesions that were solid, and 78.6% revealed a bronchus sign. The majority (88.3%) of the lesions were located in between third and fifth airway generations. That is, for lesions >20 mm, solid lesions, lesions with bronchus sign on CT images, and lesions located less than or equal to fifth airway generation, NM‐guided r‐EBUS were preferred in our center. Forceps biopsy was more frequently used (94.2%). Four lesions (3.9%) were located in fourth or fifth generation bronchus and showed “adjacent” in r‐EBUS images that were sampled by cryobiopsy, and only brush was used in two cases (1.9%) due to the “invisible” r‐EBUS images.

**TABLE 1:** Baseline characteristics of patients with peripheral pulmonary lesions.

Characteristics	Value (*N* = 102 patients)
Age, years, mean ± *SD*	64.5 ± 11.5
Sex, *n* (%)	
Male	66 (64.7)
Female	36 (35.3)
Lesion size, mm, mean ± *SD*	40.8 ± 21.7
Location of lesion, *n* (%)	
Right upper lobe	29 (28.2)
Right middle lobe	15 (14.6)
Right lower lobe	22 (21.4)
Left upper lobe	21 (20.4)
Left lower lobe	16 (15.5)
Distance from lesion to pleural, mm, mean ± *SD*	10.1 ± 12.2
Lesion characteristics, *n* (%)	
Solid	85 (82.5)
Mixed GGO	14 (13.6)
Pure GGO	4 (3.9)
With bronchus sign on CT, *n* (%)	81 (78.6)
Airway generation, *n* (%)	
Second generation	7 (6.8)
Third generation	33 (32.0)
Fourth generation	35 (34.0)
Fifth generation	23 (22.3)
Sixth generation	3 (2.9)
Seventh generation	2 (1.9)
r‐EBUS findings, *n* (%)	
Within	65 (63.1)
Adjacent	35 (34.0)
Invisible	3 (2.9)
Type of anesthesia method, *n* (%)	
General anesthesia	87 (85.3)
Moderate sedation	15 (14.7)
Biopsy method, *n* (%)	
Forceps	97 (94.2)
Cryobiopsy	4 (3.9)
Brush only	2 (1.9)
Complication, *n* (%)	
Moderate bleeding	2 (1.9)
Severe or life‐threatening bleeding	0
Pneumothorax requiring drainage	2 (1.9)

Abbreviations: CT, computed tomography; GGO, ground glass opacities.

### 3.2 Diagnostic outcomes of the included cases

**FIGURE 4:**
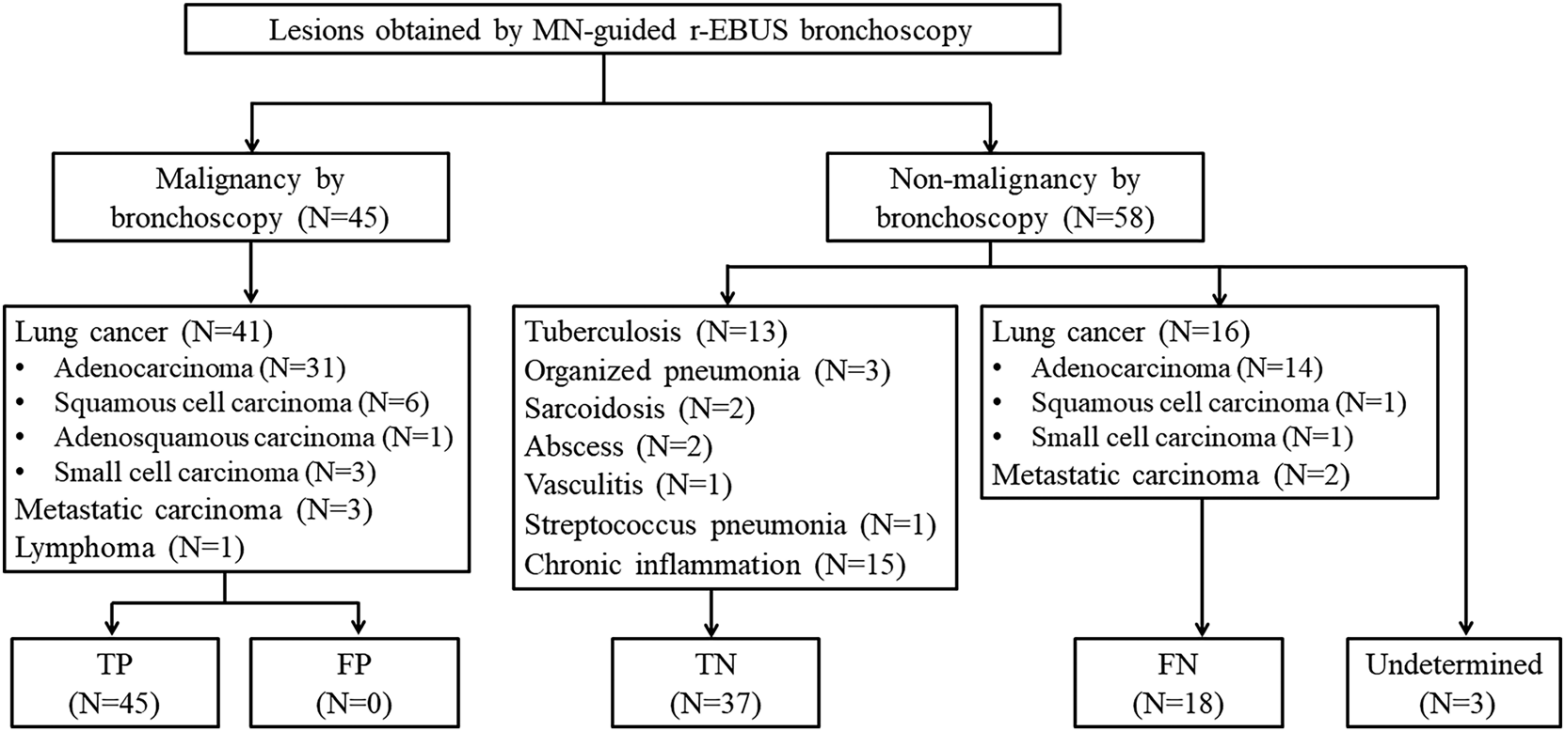
The diagnostic outcomes of the 103 lesions sampled by manual navigation (MN) guided radial endobronchial ultrasound (r‐EBUS) bronchoscopy. FN, false negatives; FP, false positive; TN, true negatives; TP, true positive.

Figure 4 depicts the diagnostic outcomes of the 103 lesions sampled by MN‐guided r‐EBUS bronchoscopy. Forty‐five lesions (43.7%) were diagnosed as malignancies, of which 41 lesions were confirmed lung cancer, three lesions were metastatic carcinoma, and one case was lymphoma. Therefore, the true positive cases for malignancy were 45, and the false positive case was zero. The remaining 58 lesions (56.3%) were initially diagnosed as nonmalignancies, of which 37 cases were confirmed true negatives, including tuberculosis (13 cases), organized pneumonia (three cases), sarcoidosis (two cases), abscess (two cases), vasculitis (one case), streptococcus pneumonia (one cases), and chronic inflammation (15 cases). Eighteen of the 58 lesions were false negatives, and they were finally diagnosed as malignancies by rebiopsy or surgery, including 16 cases of lung cancer and two cases of metastatic carcinoma. The other three cases were still undetermined after at least 6 months of follow‐up.

### 3.3 Diagnostic efficacy of MN‐guided r‐EBUS bronchoscopy

As shown in Table 2, the overall diagnostic yield of MN‐guided r‐EBUS bronchoscopy for PPLs was 82.0%, calculated as (true positive + true negative) divided by all cases, excluding the three undermined cases. The overall diagnostic yield ranged from 79.6% to 82.5%, assuming the undermined cases were all positive cases (79.6%) or negatives (82.5%). The yield for lesions >20 mm was 86.4%, ranging from 85.4% to 86.6%. The sensitivity of MN‐guided r‐EBUS bronchoscopy for malignancy was 71.4%, ranging from 68.2% to 71.4%, the specificity was 100%, the positive predictive value was 100%, and the negative predictive value was 67.3%, ranging from 63.8% to 69.0%.

**TABLE 2:** Diagnostic yield of manual navigation radial endobronchial ultrasound bronchoscopy.

	Excluding undetermined lesions (*N* = 100)	Low estimate (*N* = 103)	High estimate (*N* = 103)
Overall diagnostic yield [(TP + TN) / all lesions]	82.0% (82/100)	79.6% (82/103)	82.5% (85/103)
Sensitivity for malignancy	71.4% (45/63)	68.2% (45/66)	71.4% (45/63)
Specificity for malignancy	100% (37/37)	100% (37/37)	100% (40/40)
Positive predictive value	100% (45/45)	100% (45/45)	100% (45/45)
Negative predictive value	67.3% (37/55)	63.8% (37/58)	69.0% (40/58)

Abbreviations: TN, true negatives; TP, true positive.

### 3.4 Relation of airway generation to navigation success rate and diagnostic yield

One hundred lesions with a definite diagnosis were included to analyze the relation of airway generation to navigation success rate and yield. Table 3 showed that the diagnostic yield significantly decreased as airway generation increased, while the navigation success remained stable, and the overall navigation success rate was as high as 97.0%.

**TABLE 3:** Relation of airway generation to navigation success rate and diagnostic yield.

	Airway generation						*P*‐value
	2 (*n* = 7)	3 (*n* = 33)	4 (*n* = 33)	5 (*n* = 22)	6 (*n* = 3)	7 (*n* = 2)	
Navigation success rate (%)	7/7 (100)	33/33 (100)	32/33 (97.0)	21/22 (95.5)	2/3 (66.7)	2/2 (100)	0.155
Diagnostic yield (%)	7/7 (100)	31/33 (93.9)	27/33 (81.8)	16/22 (72.7)	1/3 (33.3)	0/2 (0)	0.003

### 3.5 Factors affecting diagnostic yield

We further investigated the factors affecting the diagnostic yield through univariate and multivariate analyses (Table 4). Univariate analysis indicated that high diagnostic yield was associated with solid lesions (OR = 3.5, 95% CI: 1.1–11.5, *P* = 0.037), with bronchus sign on CT (OR = 14.4, 95% CI: 4.4–47.0, *P* = 0.00001), and “within” image in r‐EBUS (OR = 5.9, 95% CI: 1.9–18.4, *P* = 0.002), while when the lesion was located greater than or equal to fifth airway generation, the diagnostic yield was lower (OR = 0.2, 95% CI: 0.1–0.6, *P* = 0.004). Those variables with *P*‐values less than 0.2 were entered into multivariate logistic regression model. The analysis showed that “bronchus sign on CT” was the only predictor of the overall diagnostic yield (OR = 11.5, 95% CI: 1.9–70.9, *P* = 0.009).

**TABLE 4:** Factors affecting diagnostic yield.

Variables	Univariate analysis		Multivariate analysis	
	OR (95% CI)	*P*‐value	OR (95% CI)	*P*‐value
Age	1.0 (0.9–1.0)	0.541		
Female (vs. male)	0.6 (0.2–1.7)	0.356		
Lesion size	1.3 (1.0–1.7)	0.076	0.7 (0.4–1.1)	0.096
Middle or lower lobe distribution (vs. upper lobe)	0.8 (0.3–2.3)	0.739		
Distance from lesion to pleural	0.8 (0.5–1.1)	0.138	0.7 (0.4–1.1)	0.123
Solid (vs. nonsolid)	3.5 (1.1–11.5)	0.037	2.6 (0.6–11.3)	0.188
With bronchus sign on CT (vs. without bronchus sign)	14.4 (4.4–47.0)	0.00001	11.5 (1.9–70.9)	0.009
General anesthesia (vs. moderate sedation)	1.5 (0.3–7.4)	0.612		
≥5th airway generation (vs. <5th airway generation)	0.21 (0.1–0.6)	0.004	0.2 (0.0–1.1)	0.061
“Within” in r‐EBUS (vs. adjacent or invisible)	5.9 (1.9–18.4)	0.002	1.7 (0.3–10.7)	0.591
Forceps (vs. cryobiopsy or brush)	2.4 (0.4–14.5)	0.327		

Abbreviations: CI, confidence interval; CT, computed tomography; OR, odds ratio; r‐EBUS, radial endobronchial ultrasound.

## 4 DISCUSSION

The challenge with bronchoscopic sampling of PPLs is how to navigate to the target. r‐EBUS only confirms arrival, and its diagnostic yield was only 70.6% to 72.0%, according to two large meta‐analyses.^5^, ^13^ Thus, various bronchoscopic navigation devices have been developed, such as VBN and ENB. Our study herein showed that MN achieved a high navigation success rate and overall diagnostic yield, 97% and 82%, respectively, and the sensitivity, specificity, positive predictive value, and negative predictive value for malignancy were 71.4%, 100%, 100%, and 67.3%, respectively. Moreover, the actual bronchoscopic image was almost the same as our sketch map in each airway generation (Figure 2), as Kurimoto et al. had demonstrated.^7^ Compared with VBN, MN achieved similar diagnostic yield, while MN remarkably shorten the time for planning pathway and reduced the operating cost.^10^ NAVIGATE,^14^ a prospective, multicenter, single‐arm, pragmatic cohort study on ENB, reported a diagnostic yield of 67.8%. Notably, it included nearly 50% of lesions less than 20 mm and data from general communities. Another prospective, multicenter, randomized controlled clinical trial reported that ENB‐guided r‐EBUS with a GS improved the ability to locate PPLs, achieving a diagnostic yield of 82.9%.^15^ However, both VBN and ENB heavily rely on the CT images transferred to the navigation system. Discrepancy between the inspiratory and expiratory phase‐CT scans, CT measurement algorithm errors, airway structural variation, and sputum blocking could commonly give rise to the deviation of planned path in navigation system, and at this point, manual segmentation is needed.^10^, ^16^, ^17^ A sophisticated bronchoscopist can recognize the bronchi and draw the MN map by repeatedly reading three‐dimensional multiplanar reconstruction CT images. More importantly, truly knowing and understanding the bronchial pathway will absolutely be helpful for the bronchoscopist to find the target lesion faster. Additionally, not all bronchoscopy centers can afford the expensive navigation systems.

Consistent with the previous study,^9^ the navigation success rate of MN was not affected by airway generation, because an experienced bronchoscopist could recognize the peripheral bronchi and draw the correct MN map by reading CT images. However, as the airway generation increased, the difficulty of the biopsy increased, and the diagnostic yield decreased. Because of the thickness and stiffness of the tip of the biopsy device, it may be hard to crawl up to the further bronchi with large angles^12^ and may result in displacement of the GS. Moreover, forceps must be placed almost perpendicular to the tissue to obtain a good specimen, which is quite difficult in a narrower lumen. The use of bidirectional guiding device and fluoroscopy,^9^ as well as combination of cryobiopsy and conventional sampling methods,^12^ might bring a higher yield.

The univariate analysis in our study indicated that solid lesions, the presence of bronchus sign, and “within” in r‐EBUS image were associated with higher diagnostic yields, and PPLs located greater than or equal to fifth airway generation were negatively related to clinical yields. Kho et al.^9^ also determined the factors affecting the diagnostic yield of MN‐guided r‐EBUS and reported a significant higher yield in lesions located less than fifth airway generation. Similarly, the presence of bronchus sign and “within” in r‐EBUS image were reported to be positively related to the yields according a recent large meta‐analysis.^13^ However, bronchus sign was the only significant factor associated with a higher diagnostic yield according to the multivariate analysis (OR = 11.5, 95% CI: 1.9–70.9, *P* = 0.009). The potential collinearity of the variables and insufficient sample size might contribute to these discrepancies between univariate analysis and multivariate analysis. Consistent with our multivariate analysis, the prospective study NAVIGATE^14^ on ENB reported that bronchus sign presence was the significant multivariate predictor of higher diagnostic yield, and so did ROSE. Interestingly, a meta‐analysis revealed that ROSE was the only factor associated with increased sensitivity of r‐EBUS^5^; nevertheless, the result should be interpreted with caution because of the heterogeneity between studies. Additionally, previous studies indicated that the lesion size influenced the diagnostic yield, no matter what guidance device was used.^13^, ^18^, ^19^ The large meta‐analysis on r‐EBUS also found that 20 mm was a cut‐off value for a higher yield, and the diagnostic yield was 60.5% and 75.7% for lesions ≤20 and >20 mm, respectively.^13^ However, our study failed to exhibit this relation, and it might be due to the selection bias, with 79.6% of the lesions more than 20 mm. We achieved a high diagnostic yield as 82% in this study might mainly because of the bronchus sign in the majority (78.6%) of the lesions and ROSE used in all cases, and 79.6% of the lesions were more than 20 mm.

The complication rate of MN‐guided r‐EBUS bronchoscopy was as low as 1.9% each for moderate bleeding and pneumothorax requiring drainage, and there was no severe or life‐threatening bleeding.

There are limitations in our study. First, the results from a single‐center, limited sample size, retrospective study could not be generalized to other centers. Second, there are no uniform criteria for patient selection and operation in our actual clinical practice. Third, no control group was adopted to directly compare with MN‐guided r‐EBUS. Then, though MN is an efficient and economical navigation method, its successful use requires long‐term study and repeated practice, and the diagnostic yield is related with the experiences of the bronchoscopist. Lastly, MN is an ancient and commonly known navigation method, but its usage has not been fully documented, and our data indicate that MN is still feasible nowadays, especially for PPLs with bronchus sign on CT. Further studies focused on the comparison of MN and novel navigation methods are expected.

## 5 CONCLUSIONS

Our study added valuable information on the use of MN, of which limited data are available to date, and indicated that MN‐guided r‐EBUS might be an efficient and safe diagnostic performance for PPLs, and the presence of bronchus sign in CT images is a predictor of high yield. Further large‐scale, prospective, controlled studies are needed to validate the use of MN as a guidance method.

## AUTHOR CONTRIBUTIONS

M. L. Y. and Y. X. conceptualized the study; Y. X. and M. L. Y. draw the manual navigation sketches; Y. X., L. C. W., and Y. H. performed the bronchoscopies; L. C. W., M. L. Y., and W. Y. recruited the patients and collected data; M. L. Y. and Y. X. were responsible for data curation; M. L. Y. and Y. H. drafted the original manuscript; and Y. X. reviewed and edited the draft. All authors read and approved the final manuscript.

## CONFLICT OF INTEREST STATEMENT

The authors declare that they have no competing interests.

## ETHICS STATEMENT

The study was approved by the Institutional Review Board of Hubei Public Health Clinical Center, the central hospital of Wuhan (Y‐SWJW‐LH‐2021(13)).

## Data Availability

The data that support the findings of this study are available from the corresponding author upon reasonable request.
